# The effect of copartisan justice ministers on human rights in presidential democracies

**DOI:** 10.1371/journal.pone.0234938

**Published:** 2020-09-02

**Authors:** Joshua Holzer

**Affiliations:** Westminster College, Fulton, MO, United States of America; University of Glasgow, UNITED KINGDOM

## Abstract

A body of literature suggests that states with independent courts are more likely to protect human rights. A recent article challenges this notion by arguing that when both the president and his or her justice minister share the same party—i.e., they are copartisans—that state is less likely to protect human rights, as justice ministers may value their loyalty to the president over their duty to enforce court decisions. In this article, I estimate government respect for human rights accounting for both copartisan justice ministers and an independent judiciary. In the end, I find copartisan justice ministers to be negatively associated with high government respect for human rights, even after controlling for judicial independence. Many constitutions already seek to ensure an independent judiciary, but if copartisan justice ministers increase the likelihood that governments repress, then perhaps constitutional engineers should also consider options that would reduce the likelihood that both the president and his or her justice minister share the same party.

## Introduction

In a recent article, I warn “of the human rights repercussions when one party controls both the presidency and the ministry of justice” ([1]: 8), as I find that when both the president and his or her justice minister share the same party—i.e., they are copartisans—that state is *less* likely to protect human rights. According to the literature, “independent courts constrain human rights abuses” ([2]: 1); however, this literature does not account for any negative effect resulting from copartisan justice ministers, despite the role justice ministers play in administering court decisions. Similarly, while examining the influence of copartisan justice ministers on government respect for human rights, my recent article fails to account for the potentially positive effect of an independent judiciary. The purpose of this article is to address these omissions, and towards that end, I have re-estimated my previous model—this time controlling for independent courts. In the end, I *still* find copartisan justice ministers to have negative repercussions on the likelihood that a government respects human rights.

## Background

### *De jure*, *de facto*…does it matter?

Scholars have long emphasized the important role that an independent judiciary plays not only in the functioning of a legal system [3], but also in the implementation of human rights legislation [4]. An inverse relationship between judicial independence and human rights violations is now widely known to exist [5, 6]. Regimes that protect human rights are generally found to be associated with independent courts [7], and human rights are expected to be *least* protected in the countries where the courts are *most* dependent [8, 9]. Raz ([10]: 185) argues that “[t]he rules concerning the independence of the judiciary…are designed to guarantee that they will be free from extraneous pressures and independent of all authority save that of the law,” and as such, the rules “are, therefore, essential for the preservation of the rule of law.” It is important to note, however, that there are conceptual and operational difficulties when defining ‘the rule of law’ [11]. Defining and measuring the ‘independence’ of the judiciary can be just as murky [12]. Typically, the concept of judicial independence is broken down into two subcategories: *de jure* independence and *de facto* independence.

*De jure* independence refers to the formal protections for insulating the judiciary, such as the method of appointing judges and the security of their tenure. Some even argue that judicial independence also requires budgetary control [13], for “[a]s long as the Congress controls the purse strings, members of the Supreme Court will not be totally autonomous” ([14]: 146). These safeguards are often outlined within a country’s constitution, in an attempt to prevent other branches from tilting the balance of power in their favor. While Cross [5] finds significant linkages between *de jure* independence and improvements in human rights practices, Keith [15] argues that such linkages are not nearly as encouraging as one would hope. In her examination of “the impact of constitutional provisions…on state abuse of the right to personal integrity,” she finds that “none of the constitutional provisions for individual freedoms is statistically significant” ([15]: 111). Such results add to a growing chorus that argues that *de jure* “judicial independence alone does not ensure that the courts will play an active role in protecting rights” ([16]: 29).

Many scholars now argue that the disconnect between *de jure* judicial independence and the expectation of better human rights practices can be explained by the difference between *de jure* and *de facto* independence. While *de jure* independence refers only to formally written protections, *de facto* independence “is often defined as the extent to which a court may adjudicate free from institutional controls, incentives, and impediments imposed or intimidated by force, money, or extralegal, corrupt methods” ([17]: 286). As Melton and Ginsburg ([18]: 209) point out, “clever politicians can exploit the absence of any single *de jure* protection for judicial independence.” “This,” they argue, “helps us understand why, over the last 25 years, increases in *de jure* protection of judicial independence have not been followed by similar increases in *de facto* judicial independence” ([18]: 210).

So whereas *de jure* independence has not been reliably found to equate to high government respect for human rights, Howard and Carey ([17]: 290) suggest that *de facto* independence “leads to greater political rights.” The challenge, however, is that *de facto* judicial independence “is not directly observable” ([19]: 107). In an attempt to address this, Linzer and Staton [20] have constructed a new latent *de facto* measure of judicial independence that draws upon previous direct and indirect indicators by Keith [21], Howard and Carey [17], Feld and Voigt [22], Gwartney *et al.* [23], Cingranelli and Richards [24], Marshall and Jaggers [25], Johnson, Souva, and Smith [26], and the Political Risks Services Group [27]. Using Linzer and Staton’s [20] measure, both Crabtree and Fariss [2] and Crabtree and Nelson [28] have found *de facto* judicial independence to be positively associated with high government respect for human rights. Unfortunatley, independent courts only matters to the extent that the executive branch is willing to *enforce* that court’s decision; neither Crabtree and Fariss [2] nor Crabtree and Nelson [28] take this into account. As such, I argue that in addition to focusing on the *de facto* independence of a court, scholars should *also* be looking at the ‘independence’ of the executive authority charged with implementing court decisions.

### Copartisans and court decisions

As many have noted, government policies implemented as a result of court decisions are not always a perfect translation of those decisions [29–32]. This is because “[j]udicial decisions are not self-executing” ([33]: 27). In the Federalist Papers, Hamilton ([34]: 239) argues that “the independence of the judges may be an essential safeguard against the effects of occasional ill humors in the society,” yet Hamilton ([34]: 236) also readily acknowledges that courts have “neither FORCE nor WILL, but merely judgment; and must ultimately depend upon the aid of the executive arm even for the efficacy of its judgments.” Building upon this idea, Spriggs ([35]: 1122-1123) suggests that the “imperfect translation of Court opinions into agency policies” is *deliberate*, as sitting governments “are strategic and implement Supreme Court opinions based upon their expectations of the costs and benefits.” Typically, the ministry of justice is charged with administering justice, and the justice minister is legally bound to follow court decisions and implement them accordingly. However, while courts are ideally independent, justice ministers are usually selected by—and answerable to—a partisan executive. As such, justice ministers are often appointed as a result of party loyalty. Can justice really be blind when wearing partisan blinders?

In my recent article [1], I find that when both the president and his or her justice minister share the same party, that state is *less* likely to protect human rights. Why is this the case? First, a copartisan justice minister could aid a president that wishes to repress rights. Second, a repressive justice minister may find themselves less constrained by a copartisan president. Take, for example, the case of United States (US) President Andrew Jackson and Attorney General Roger B. Taney.

In 1831, Worcester and several other non-Native American missionaries were indicted in the US state of Georgia under an 1830 act of the Georgia legislature meant “to prevent white persons from residing within that part of the chartered limits of Georgia occupied by the Cherokee Indians…and to enforce the laws of the state within the aforesaid territory” ([36]: 539). Worcester was ultimately convicted and “condemned to hard labour for four years in the penitentiary of Georgia” ([36]: 536). In an opinion delivered by Chief Justice John Marshall, the US Supreme Court held that the Georgia act, under which Worcester had been prosecuted, violated the US Constitution, treaties between the US and the Cherokee Nation, and “an act [of the US Congress] to regulate trade and intercourse with the Indian tribes” ([36]: 540). In essence, the Georgia act interfered with the federal government’s authority and was deemed unconstitutional. Upon hearing of Marshall’s decision, President Jackson—who sided with Georgia in this confrontation and opposed Native American sovereignty—is alleged to have said: “John Marshall has made his decision: *now let him enforce it!*” ([37]: 384).

Today, Jackson is derided for his role in tramping the rights of Native Americans and supporting slavery [38], yet Jackson was aided in these endeavors by a loyal attorney general, “political hack, Roger B. Taney” ([39]: 554). At this time it is important to note that in some countries, government ‘ministries’ are called *departments* (e.g. the US Department of Justice). Furthermore, note that in some countries, the ‘justice minister’ is called the minister *of* justice, the minister *for* justice, the *secretary* of justice, or in some cases (like in the US) the *attorney general*. For the sake of simplicity, I simply use the generic term ‘justice minister’ throughout this article, except for this US anecdote. Continuing on, in his Pulitzer-Prize winning study on Jackson, Schlesinger ([40]: 65) refers to Taney as Jackson’s “spearhead of radicalism.” In addition to fighting Native American sovereignty and supporting slavery, Finkelman ([41]: 92) notes that “as attorney general, Taney had believed that free blacks had no rights that any government had to protect.” Calhoun ([42]: 612) adds that “Taney—as he sometimes did—found in the law what he wanted to find.” Ultimately, Taney’s “obsessive fidelity to Jacksonian tenets” was later rewarded, when Jackson appointed him to the Supreme Court as Chief Justice, upon Chief Justice Marshall’s death ([39]: 584). Taney was promoted to replace the man that he had been complicit in helping Jackson ignore! As illustrated in this particular case, a copartisan ‘justice minister’ worked as an enabler to a president determined to trample rights (even in opposition to an independent Supreme Court).

What about when the justice minister is *not* a copartisan? First, a repressive justice minister that is *not* a copartisan may find themselves more constrained by the president, and therefore less likely (and/or able) to repress rights. Second, a copartisan justice minister could ‘sound the alarm’ in the face of a president that is repressing (or wishes to repress) rights. For instance, if a president were to propose a new policy (or the renewal of a current policy) that the justice minister disagreed with, the justice minister could attempt to effectively ‘veto’ said policy by privately—or publicly—threatening to withdraw their support of the president. The justice minister could also attempt to exercise autonomy over how (or *whether*) the president’s policy is implemented. The president, if even aware of the insubordination, would then be in the awkward position of having to sack the justice minister or ask for their resignation, which often has a political cost. Take, for example, the case of Indonesian President Abdurrahman Wahid and Justice Minister Yusril Mahendra.

In 1999, Wahid became Indonesia’s first elected president after the end of Suharto’s 30-year rule. Wahid’s presidency started with high hopes, but soon after coming to power “[h]is erratic and amateur approach to administration” ([43]: 273) led to a series of missteps. To make matters worse, “[h]e also interfered with legal processes” ([44]: 95), and as a result, “[h]ostile parliamentarians accused him of a broad array of misdemeanours and failings” ([43]: 273). In response, Wahid “threatened to jail his political opponents,” thereby revealing “an authoritarian tendency” that would become increasingly apparently throughout his presidency ([44]: 95).

By 2001, Indonesian’s parliament began considering the impeachment of Wahid, and to preempt this, “Wahid threatened to ‘freeze’ the parliament” ([44]: 95). In response, “[t]he Supreme Court ruled that Wahid lacked the constitutional power to dissolve the legislature” ([45]: 31), which is effectively what a ‘freeze’ would have been. During this time, “Justice Minister Yusril Ihza Mahendra was sacked for making repeated public calls for the president to resign” ([46]: 354). Mahendra—who was *not* a copartisan—had previously suggested that Wahid resign during a private cabinet meeting earlier in the year ([46]: 350). When the suggestion was rebuffed, Mahendra began speaking out in public. Mahendra’s sacking by Wahid eventually led to a ministerial revolt, at which point most of those that had once supported Wahid’s “presidential bid turned against him” ([47]: 124). Ultimately Wahid was successfully impeached. If Mahendra *had* been a copartisan, he might have been less willing to support Wahid’s removal; instead, Mahendra put his job as justice minister in jeopardy by leading the charge against Wahid. As illustrated in this particular case, a justice minister that was *not* a copartisan chose loyalty to the law over loyalty to an authoritarian president.

To review, some argue that an independent judiciary promotes high government respect for human rights; however, these studies fail to account for any negative effect resulting from copartisan justice ministers. In a recent article, I argue that country-years where both the president and the justice minister are of the same party are more likely to be associated with poor human rights practices; however, my study fails to account for the potentially positive impact of an independent judiciary. This leads me to the primary question *this* article seeks to address:

Are copartisan justice ministers *still* negatively associated with high government respect for human rights when one controls for the independence of the judiciary?

## Methods

### Sample

As my just stated research question suggestions, the purpose of this article is to ascertain whether copartisan justice ministers are *still* negatively associated with high government respect for human rights when one controls for the independence of the judiciary. This idea seeks to extend the findings of my previous article [1], which first examined the relationship between copartisan justice ministers and government respect for human rights in presidential democracies. In order to build upon my previous empirical findings, my sample is modeled of that which was used in my previous study—i.e. presidential democracies. While many early human rights studies have utilized either the Polity or Freedom House measures to identify which regimes are democratic, this is problematic as both Polity and Freedom House classify regimes—in part—based on their respect for human rights, and therefore, using either measure would partially control for my outcome variable [2]. As such, following the trend of more recent human rights studies (e.g. [1, 2, 28, 48]), I utilize Cheibub, Gandhi, and Vreeland’s Democracy versus Dictatorship dataset [49] to determine what constitutes a ‘democracy’. Per their classification, a regime is considered to be a ‘democracy’ when the president is elected, the legislature is elected, there is more than one party competing in elections, and an alternation under identical electoral rules has taken place ([49]: 69). Consistent with my previous study [1], I utilize Bormann and Golder’s Democratic Electoral Systems Around the World dataset [50] to further narrow my pool of democracies to specifically ‘presidential’ democracies.

Why only presidential systems? Practically, I am limited to those particular country-years until such as time as more data becomes available. Theoretically, this makes sense anyway, as there are important differences between presidential and parliamentary systems, particularly with regard to judicial matters. For instance, Samuels and Shugart ([51]: 255) note that in presidential systems, “the separation of powers creates presidentialized parties.” Presidents “often engineer a *de facto* reversal of the party-executive principal-agent relationship” found in parliamentary systems, and as such, presidents “come to control parties for their own purposes” ([51]: 251). In other words, presidents may have more influence over copartisans than prime ministers.

Furthermore, Moreno, Crisp, and Shugart ([52]: 89) argue that *de facto* judicial independence can only exist in democratic “presidential systems, where there is no notion of parliamentary sovereignty,” as only in these countries do “courts typically have authority that overlaps with the elected bodies and may even overturn acts of the elected bodies on constitutional grounds.” In essence, this line of argument asserts that parliamentary sovereignty is often incompatible with judicial review, which itself is a requirement for *de facto* judicial independence. “Courts are independent to the extent that they are not accountable to the political bodies” ([52]: 90). When courts are *not* empowered to overturn laws—as is the case in many parliamentary systems—such courts are “essentially another part of the bureaucracy,” subservient to those doing the legislating ([52]: 89). This echoes Hamilton’s ([34]: 237) fear that if “the legislative body are themselves the constitutional judges of their own powers,” this could “enable the representatives of the people to substitute their WILL to that of their constituents.” However, “[i]n presidential systems…[c]ourts that have the authority to veto legislative acts are thus another actor in the process” ([52]: 90). In such systems, an independent judiciary serves as “an intermediate body between the people and the legislature, in order, among other things, to keep the latter within the limits assigned to their authority” ([34]: 237).

Given data limitations, given the impact that ‘presidentialized’ parties may have on the relationship between presidents and copartisans, and given the importance of judicial review (which is not present in all parliamentary systems) to *de facto* judicial independence, both practically and theoretically it seems appropriate to limit my sample to presidential systems. As a result, however, note that my findings cannot likely be generalized *beyond* presidential democracies. With that said, although my sample size is limited to only about a decade, I see no reason why my findings cannot also be applicable to presidential democracies in earlier or more recent decades.

### Dependent variables

For my primary dependent variable, I utilize the Cingranelli-Richards Physical Integrity Rights Index (CIRI Index), which is an additive nine-point index of the following four ordinal indicators of government respect for physical integrity rights: the right to be protected from political imprisonment, torture, disappearance, and extrajudicial killing [53]. *CIRI Index* scores ranges from ‘0’ (no respect for any of the four physical integrity rights) to ‘8’ (full respect for all of them). I also utilize an alternative dependent variable: the Political Terror Scale (PTS), which—like the *CIRI Index*—is coded using data from the US Department of State’s Country Reports on Human Rights Practices and Amnesty International’s Annual Report [54]. While *CIRI Index* scores are determined by *individually* evaluating instances of political imprisonment, torture, disappearance, and extrajudicial killing (then adding together all four constituent scores), *PTS* scores are determined by *collectively* evaluating the range of the population effected by instances of political imprisonment, torture, disappearance, and extrajudicial killing. Countries are designated a level ranging from ‘1’ to ‘5’: ‘1’ indicates that the state is under a secure rule of law, people are not imprisoned for their views, torture is rare or exceptional, and political murders are extremely rare; ‘2’ indicates that there is a limited amount of imprisonment for nonviolent political activity, torture is exceptional, and political murder is rare; ‘3’ indicates that there is extensive political imprisonment, and political murders are common; ‘4’ indicates that disappearances, torture, and political murders are all common, though state terror only affects those who interest themselves in politics; finally, ‘5’, which indicates that state-sanctioned repression has been extended to the whole population, and state leaders place no limits on the means or thoroughness with which they pursue personal or ideological goals [55].

While *CIRI Index* scores aim to reflect “actual government practices” ([24]: 406), *PTS* scores aims to reflect “the ‘range’ of violence committed” ([55]: 368). Despite these differences, however, “PTS and CIRI essentially measure the same thing” ([56]: 88). As such, scholars that employ one of these indices as their dependent variable often report analogous estimations using the alternate index as a robustness check (e.g. [48, 57–59]). In order to aid in comparability to the *CIRI Index*, I have followed the literature’s trend by inverting *PTS* scores, such that higher scores now indicate greater government respect for physical integrity rights.

Despite their widespread use, in recent years *CIRI Index* scores and *PTS* scores have had to contend with some amount of criticism. For instance, Clark and Sikkink ([60]: 567) argue that “[b]ecause of increased quality and quantity of information,” both *CIRI Index* and *PTS* scores “skew toward worse scores in later years.” Building upon this idea, Fariss ([61]: 300) argues that over time, “the U.S. State Department and Amnesty International look harder for abuse, look in more places for abuse, and classify more acts as abuse.” He calls “this process…a *changing standard of accountability*” ([61]: 297). To address this, he has created new latent scores for human rights which he claims are “unbiased estimates of repression” ([61]: 297). He then uses his scores to “show that respect for human rights has improved over time,” which seemingly supports the argument that *CIRI Index* scores and *PTS* scores are skewed downward as a result of more critical US State Department and Amnesty International reporting in recent years. As a result, some scholars have abandoned the use *CIRI Index* scores and/or *PTS* scores in favor Fariss’ latent human rights scores.

In response, Haschke and Gibney ([56]: 89–90) note that Clark and Sikkink [60] and Fariss [61] “repeatedly assert that the [US State Department and Amnesty International] annual reports are now longer and more detailed—and presumably more accurate—than they had been in the past,” yet “the evidence that they marshal in this regard is selective and does not constitute compelling proof.” In their analysis, Haschke and Gibney ([56]: 99) show that “any measurable bias is actually in the opposite direction of” what Clark and Sikkink and Fariss claim. In another response, Cingraneli and Filippov ([62]: 1088) note that they “have identified serious problems” with the alleged ‘unbiased estimates’ created by Fariss. Using Fariss’ own computer code they find that the upward trend identified by Fariss “depended almost entirely on the inclusion of the mass killing indicators” ([62]: 1086). They caution that “[t]hose who use Fariss’s scores should be aware that there is a strong built-in correlation between mass killings and those scores,” and as such, “evaluators should remember that the trends in Fariss’s scores for capable and democratic countries are affected by frequencies of mass killing events in failed and authoritarian states” ([62]: 1088). For this reason alone, it would be inappropriate for me to use Fariss’ scores as my sample is limited to democracies. However, Cingraneli and Filippov ([62]: 1089) also caution “that latent scores should not be used as dependent variables in conventional regression analysis because doing so could produce inconsistent or severely biased estimates.” To further drive the point home, they argue that in creating his scores, Fariss “comes close to data manufacture, and scores fabricated in this way should not be used in place of carefully collected and consistently coded real human rights data” such as the *CIRI Index* and *PTS* ([63]: 274). An even more recent article [64] further calls into question the validity of Fariss’ scores.

In short, although some scholars have followed Fariss in utilizing his new scores, these estimates are not without controversy and—given recent critiques—they are not the most appropriate for my particular research question. On final alternative I would like to briefly touch on is the latest version of the new Varieties of Democracy (V-Dem) dataset [65]. Given that the dataset does include a few human rights measures, some [66] has suggested that it could be used as an alternative to *CIRI Index* scores and *PTS* scores. While this new version shows promise, *V-Dem*’s Physical Violence Index (which would be the measure most comparable to *CIRI Index* scores and *PTS* scores) is only an aggregation of their extrajudicial killings and torture indicators. Note that unlike *CIRI Index* scores and *PTS* scores, *V-Dem*’s Physical Violence Index does *not* account for either disappearances or political imprisonment. In other words, *CIRI Index* scores and *PTS* scores are much more similar to one another than either are to *V-Dem*’s Physical Violence Index, and as such, I argue that if my primary dependent variable is *CIRI Index* scores, *PTS* scores would be a more appropriate check for robustness than *V-Dem* scores. To conclude, I would like to point out that neither *CIRI Index* scores nor *PTS* scores are relics of some bygone era of human rights research; in 2020 alone, there has already been several human rights-related papers published that have opted to use these scores in place of (and despite the creation of) the new Fariss and *V-Dem* measures (e.g. [1, 67–69]).

### Independent variables

My primary independent variable—*copartisan justice minister*—is pulled from my previous article [1]. This variable is coded as ‘1’ for every country-year where the president and his or her justice minister are in the same party (and ‘0’ when they are *not* in the same party). Further details of this variable’s construction can be found in my previous article. For my *de facto* judicial independence variable, I follow others [2, 28] in using Linzer and Staton’s [20] measure. However, I use their most recently updated version [70], and for the sake of brevity, I simply refer to this variable as *judicial independence*. Beyond the *copartisan justice minister* and *judicial independence* variables, I use the *exact same* control variables as my previous article; these variables include a measure of constitutional checks on the executive, the level of civil conflict, population size, gross domestic product (GDP) per capita, and finally a control for the previous year’s level of government repression. Note that since Poe and Tate [71], the inclusion of such variables has become common practice in models that estimate government repression, and as a result of the significance of their article (and later extensions [72, 73]) many studies within the human rights literature now generically refer to similar model specifications as “Poe and Tate model[s]” ([74]: 663). At this point, I will elaborate on some specifics of my particular Poe and Tate model. Note that summary statistics are presented in Table 1.

**Table 1:** Summary statistics.

	Obs	Countries^†^	Min	Mean	Mode (Freq)	Max	Std Dev
Copartisan justice minister	441	47	0	0.472	0 (233)	1	0.500
CIRI Index^1^	441	47	0	5.202	7 (112)	8	1.903
CIRI Political Imprisonment^2^	441	47	0	1.481	2 (261)	2	0.688
CIRI Torture^2^	441	47	0	0.63	1 (206)	2	0.630
CIRI Disappearance^2^	441	47	0	1.823	2 (381)	2	0.477
CIRI Extrajudicial Killing^2^	441	47	0	1.268	2 (191)	2	0.727
PTS^3^	441	47	1	3.533	4 (160)	5	0.970
Judicial independence^4^	441	47	0.085	0.593	—	0.976	0.233
Presidential constraints^5^	441	47	0.350	0.666	—	0.970	0.158
Civil conflict^6^	441	47	0	0.120	0 (397)	2	0.383
(Logged) population size	441	47	12.601	16.242	—	19.558	1.442
(Logged) GDP per capita	441	47	6.363	9.138	—	10.840	1.008

^†^Armenia, Austria, Bolivia, Brazil, Bulgaria, Chile, Colombia, Costa Rica, Croatia, Cyprus, the Dominican Republic, El Salvador, Finland, France, Georgia, Ghana, Guatemala, Guinea-Bissau, Honduras, Iceland, Indonesia, Kenya, Lithuania, Malawi, Mali, Mexico, Mongolia, Nicaragua, Nigeria, North Macedonia, Panama, Paraguay, Peru, the Philippines, Poland, Portugal, Romania, Serbia, Sierra Leone, the Slovak Republic, Slovenia, Sri Lanka, Timor-Leste, Ukraine, the United States of America, Uruguay, and Venezuela.

^1^Higher values indicate greater government respect for human rights. Note that this an additive index of the following four indicators: the right to be protected from political imprisonment, torture, disappearance, and extrajudicial killing.

^2^Higher values indicate greater government respect for human rights.

^3^Values have been inverted such that higher values now also indicate greater government respect for human rights

^4^Higher values indicate greater *de facto* judicial independence.

^5^Higher values indicate greater constraints on the power of the president.

^6^0 indicates < 25 battle-related deaths, 1 indicates between 25 and 999 battle-related deaths, and 2 indicates > 1000 battle-related deaths

To begin, many Poe and Tate models include Polity IV’s XCONST variable as a control (e.g. [74–76]); this variable takes into account “the extent of institutional constraints on the decision-making powers of the chief executive” ([77]: 62). However, Doyle and Elgie ([78]: 732) point out that while “some studies have estimated the effect of variation in the level of constraints on the executive in the system of checks and balances by operationalizing Polity’s XCONST variable,” this measure does not fully account for all the variation *between* various presidential systems. In other words, since Polity IV’s XCONST measure was designed to estimate executive constraints across *all* countries, it does not fully capture the variation within specifically *presidential democracies*, which is the focus of this article. As such, I follow recent articles [79, 80] in using Doyle and Elgie’s ([78]: 734) quantification of “the constitutional power of presidents,” which I have inverted and refer to as *presidential constraints*. The inclusion of this variable as a control is particularly important to this article’s research question as “judges are less likely to invalidate legislation or governmental actions in countries possessing strong presidents” ([81]: 435).

Continuing on, Poe and Tate ([71]: 859) note that “regimes are more coercive when they are involved in civil conflict.” The measure of *civil conflict* I utilize is drawn from the Uppsala Conflict Data Program [82]; the use of this particular dataset is common within the human rights literature (e.g. [75, 79, 80, 83]). My *civil conflict* variable is coded as ‘0’ for each country-year with less than 25 battle-related deaths, ‘1’ for each country-year where there were between 25 and 999 battle-related deaths, and finally ‘2’ for each country-year where there were more than 999 battle-related deaths.

To conclude, Poe and Tate models typically include measures for population size (which seems to be *negatively* associated with high government respect for human rights), GDP per capita (which seems to be *positively* associate with high government respect for human rights), as well as a lag of the dependent variable (as a country’s previous year’s level of government repression seems to influence the following year’s level of government repression). First, both my population size and GDP per capita measures are from the World Bank [84], and—following the literature—both of these measures have been logged to correct their distributional nature [85]. Second, although Poe and Tate [71] include a lagged dependent variable, “[b]ecause the CIRI [and PTS] variables are non-linear, a simple lagged dependent variable is less appropriate because it does not efficiently model the autoregressive trend in the data” ([86]: 12). As such, I follow Hafner-Burton ([87]: 615) in including a series of “binary indicators measuring a state’s previous level of repression…to account for dependence across the categories of the dependent variable over time”; this particular technique has since been used in many other human rights-related studies (e.g. [48, 73, 86, 88]). Because these binary “variables are included for diagnostic rather than substantive reasons and given the large number of them,” like others, I do not report their estimates when discussing my findings ([73]: 298). However, note that they are all statistically significant, as can be seen using my replication files.

### Model specification

At this point, I would like to remind the reader that my dependent variables (i.e. *CIRI Index* scores and *PTS* scores) are both *ordinal*, and therefore, an ordinary least squares (OLS) regression (which is linear) would lead to biased inferences. Long and Freese ([10]: 309) warn that while “it is tempting to analyze ordinal outcomes with the linear regression model (LRM)…an ordinal dependent variable violates the assumptions of LRM, which can lead to incorrect conclusions.” Instead, they argue that “[w]ith ordinal outcomes, it is much better to use models that avoid the assumption that the distances between categories are equal” ([10]: 309). Following Long and Freese’s advice, the most appropriate way to test my research question would be to utilize ordered probit regression. Indeed within the broader human rights literature, models that similarly estimate *CIRI Index* scores and/or *PTS* scores commonly rely upon the use of ordered probit regression (e.g. [1, 48, 57, 58, 68, 75, 88]).

### Results

Table 2 shows that my *copartisan justice minister* variable is found to be negatively associated with both high *CIRI Index* scores and high *PTS* scores; these relationships are statistically significant at least at the 95% level. Notably, both models control for *judicial independence*, which (as expected) is found to be positively associated with both high *CIRI Index* scores and high *PTS* scores. Consistent with previous human rights scholarship, across both models all remaining statistically significant control variables have signs pointing in the expected direction. For instance, *presidential constraints* and *(logged) GDP per capita* are both found to be positively associated with both high *CIRI Index* scores and high *PTS* scores, while *civil conflict* and *(logged) population size* are both found to to be negatively associated with both high *CIRI Index* scores and high *PTS* scores.

**Table 2:** Ordered probit estimates of CIRI Index scores and PTS scores in presidential democracies, 2001-2011.

	CIRI Index	PTS
Copartisan justice minister	-0.316***	-0.255**
	(0.113)	(0.122)
Judicial independence	1.943***	1.646***
	(0.301)	(0.409)
Presidential constraints	0.679*	1.056*
	(0.376)	(0.573)
Civil conflict	-0.876***	-0.922***
	(0.187)	(0.343)
(Logged) population size	-0.220***	-0.221***
	(0.053)	(0.068)
(Logged) GDP per capita	0.169**	0.115
	(0.079)	(0.108)

* p < 0.10,

** p < 0.05,

*** p < 0.01.

Numbers in parentheses are robust standard errors clustered by country. For CIRI Index, higher values indicate greater government respect for human rights. For PTS, the values have been inverted such that higher values now also indicate greater government respect for human rights. For diagnostic reasons, both models include binary variables for each level of the dependent variable (excluding the most repressive category), lagged. These binary variables are all statistically significant at the < 0.01 level; for space considerations, they are not reported.

**Fig 1:**
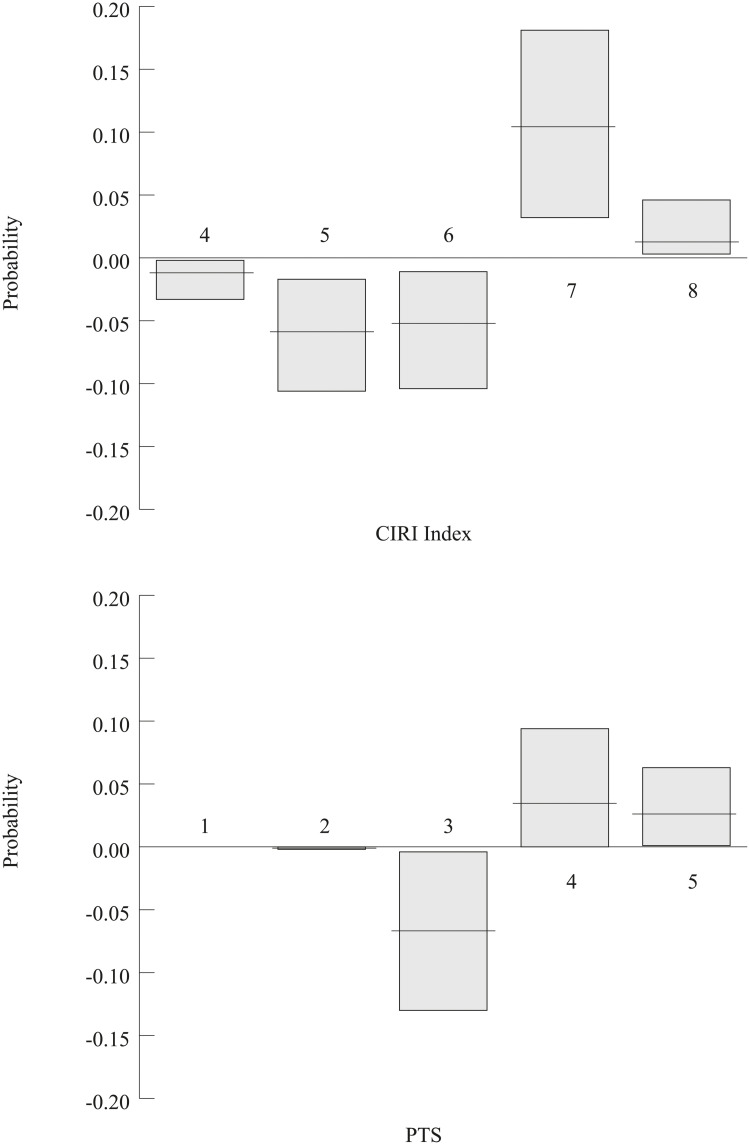
Predicted probabilities (and 95% confidence intervals) for CIRI Index scores and PTS scores when the justice minister goes from copartisan to *not* a copartisan.

In Fig 1, I illustrate predicted probabilities (and 95% confidence intervals) for *CIRI Index* scores and *PTS* scores when the justice minister goes from copartisan to *not* a copartisan. Note that these probabilities are based upon each control variables’ mean (or mode in the case of *civil conflict*, as it is categorical); this data can be seen in Table 1. These probabilities were estimated using the Clarify software package [89] and help to provide substantive meaning to the models reported in Table 2.

Starting with the top-half of Fig 1, you can see that for the ‘average’ state in my dataset (i.e. a state whose parameters match the mean/mode of my control variables), if that state were to have a justice minister that is *not* a copartisan (instead of a copartisan), the predicted probability of having a *CIRI Index* score of 7 or 8 goes *up*, while the predicted probability of having a *CIRI Index* score of 4, 5, or 6 goes *down*. Recall that higher *CIRI Index* scores indicate greater government respect for human rights, and the scale tops out at 8. While *CIRI Index* scores can go down to 0, those scores are not listed in Fig 1 as the predicted probability of such scores (as well as any change in such scores) is effectively zero. This means that for the ‘average’ state, switching from a copartisan justice minister to a justice minister that is *not* a copartisan *increases* the probability of high government respect for human rights, and *decreases* the probability of low government respect for human rights. The bottom-half of Fig 1 follows this same trend; for the ‘average’ state, if that state were to have a justice minister that is *not* a copartisan (instead of a copartisan), the predicted probability of having a *PTS* score of 4 or 5 goes *up*, while the predicted probability of having a *PTS* score of 3 goes *down*. Any change in the predicted probability of 1 or 2 is negligible. Recall that I have inverted *PTS* scores, such that higher scores (which top out at 5) now indicate greater government respect for human rights. Note that these changes I have just described are statistically significant at the 95% level (as indicated by the gray confidence intervals that do not overlap with zero).

**Fig 2:**
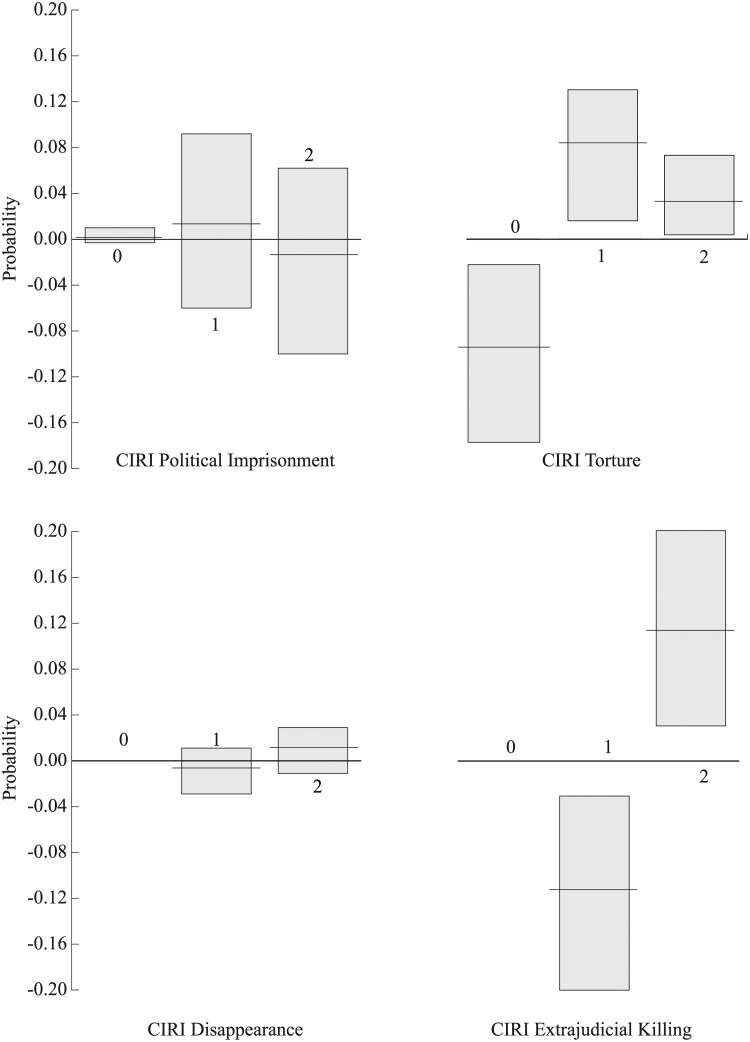
Predicted probabilities (and 95% confidence intervals) for disaggregated CIRI Index scores when the justice minister goes from copartisan to *not* a copartisan.

As previously mentioned, the *CIRI Index* is an additive index. *CIRI Index* scores are calculated by adding together *CIRI Political Imprisonment* scores, *CIRI Torture* scores, *CIRI Disappearance* scores, and *CIRI Extrajudicial Killing* scores; as can be seen in Table 1, each of these four ordinal indicators of government respect for specific physical integrity rights range from ‘0’ (which indicates poor respect) to ‘2’ (which indicates full respect). Unlike *PTS*, one of the benefits of the *CIRI Index* is that these these four ordinal indicators can be disaggregated. In Fig 2, I illustrate predicted probabilities (and 95% confidence intervals) for disaggregated *CIRI Index* scores when the justice minister goes from copartisan to *not* a copartisan. As with Fig 1, these probabilities correspond to the ‘average’ state in my dataset (i.e. a state whose parameters match the mean/mode of my control variables).

Starting with the left-side of Fig 2, note that all the gray *CIRI Political Imprisonment* and *CIRI Disappearance* confidence intervals overlap with zero, which indicates that the probability changes are *not* statistically significant (at the 95% level). This suggests that country-years with copartisan justice ministers are no more—and no less—likely to have different *CIRI Political Imprisonment* scores or *CIRI Disappearance* scores versus country-years where the justice ministers is *not* a copartisan. Moving to the right-side of Fig 2, note that the predicted probability of having a *CIRI Torture* score or *CIRI Extrajudicial Killing* score of 2 goes up when the justice minister is *not* a copartisan. Furthermore note that these gray confidence intervals do *not* overlap with zero. This suggests that country-years where the justice ministers is *not* a copartisan are more likely than country-years where the justice minister is a copartisan to have the highest *CIRI Torture* score and *CIRI Extrajudicial Killing* score. In other words, in comparison to when the justice minister and president share the same party, it appears that when the justice minister is *not* in the same party as the president, such governments are less likely to condone the use of torture and extrajudical killings.

## Conclusion

According to Hamilton ([34]: 236), “the judiciary is beyond comparison the weakest” branch of government, as it “has no influence over either the sword or the purse…and can take no active resolution whatever.” In light of Hamilton, I argue that in addition to focusing on whether *courts* are independent of undue influence, scholars should also be looking at whether *justice ministers* are independent of undue influence, given the role justice ministers play in administering court decisions. A body of literature suggests “that independent courts constrain human rights abuses” ([2]: 1), yet these studies do not take into account copartisan justice ministers. Similarly, while examining the influence of copartisan justice ministers on government respect for human rights, my recent article [1] fails to account for the impact of an independent judiciary. With this article, I have sought to remedy these omissions by re-estimating my previous model while controlling for the independence of the judiciary, and in the end, I *still* find copartisan justice ministers to be negatively associated with high government respect for human rights.

At this point, many constitutions already include provisions to ensure *de jure* judicial independence, yet—to my knowledge—no constitution prohibits the justice minister from being a copartisan of the president. Volcansek and Lockhart ([90]: 33) argue that “constitutional drafters should focus less on institutional elements related to judicial independence…[r]ather, those crafting constitutions intended to protect human rights should look to dispersing government power.” If copartisan justice ministers increase the likelihood that governments repress, then perhaps constitutional engineers should consider options that would reduce the likelihood that both the president and his or her justice minister share the same party.
